# Self-administration of a *Salmonella* vaccine by domestic pigs

**DOI:** 10.1038/s41598-023-29987-x

**Published:** 2023-02-20

**Authors:** Rebecca C. Robbins, Courtney Archer, Luis G. Giménez-Lirola, Juan Carlos Mora-Díaz, John J. McGlone

**Affiliations:** 1 R.C. Robbins Swine Consulting Services PLLC, 1113 Alta Vista St., Amarillo, TX 79106 USA; 2 Laboratory of Animal Behavior, Physiology and Welfare, Texas Tech University, Lubbock, TX 79409-2141 USA; 3 Department of Veterinary Diagnostic and Production Animal Medicine, College of Veterinary Medicine, Iowa State University, Ames, IA 50011 USA

**Keywords:** Vaccines, Animal behaviour, Immunology

## Abstract

Hand vaccinating is time consuming and inefficient. Oral vaccines delivered by drenching are less likely to be used due to a lack of labor on farms. Current environmental enrichment (EE) technologies do not allow pigs to express certain natural behaviors such as rooting and getting a reward. We developed a sprayer so that domestic pigs can self-apply any liquid. By adding an attractant (pig maternal pheromone), the use of EE devices by individual pigs can be increased. In this study, we used a *Salmonella* oral vaccine to evaluate efficacy of three delivery methods: (1) Control, no vaccine, (2) hand drenching as labeled, and (3) self-administration by this EE rooting device. All pigs sprayed themselves within 80 min of exposure to the EE device. While control pigs had little or no *Salmonella* serum and oral fluid IgG or IgA, hand-drenched and self-vaccinated pigs built similar levels of both serum and oral fluid IgA and IgG. We conclude we were able to significantly reduce human labor needed and achieved 100% efficacy in eliciting a serologic response when pigs self-administered a *Salmonella* vaccine. This technology could benefit commercial pig production while providing an enriched behavioral environment. Self-vaccination could also assist in control or immunization of feral swine and improve domestic pig health and food safety.

## Introduction

USA pig producers are experiencing a shortage in labor^1^. Rural labor markets, where pig farms generally operate, have below average unemployment. Slowed population growth in the locations where farms are located further drains the labor pool. Hog farm employment has fallen 0.3% from 2019 to 2020^1^. Labor has remained approximately 10% of cash expenses for US hog producers and is a major cost of producing a pig^2^. In response, agriculture sectors have and will need to continue increasing mechanization and seeking technological advances to reduce labor requirements while supporting animal needs^2^.

Pig producers are now seeking smart farming, digital farming, and precision farming solutions. Smart farming is applying technologies that improve efficiency without requiring an increase in resources such as labor. Current approaches for vaccine administration require significant time with intensive human-animal direct interactions^3^. This can interrupt normal behavior and be a stressful time for the animal and workers.

Environmental enrichment (EE) for commercial pigs is required in some countries and encouraged in the world-wide marketplace. Available EE devices have focused on either hanging or on-floor toys that often do not accommodate the behavioral need for pigs to root and push/dig. Most awake and active behaviors of pigs involve rooting and other oral/facial/nasal behaviors (ONF) regardless of the housing system^4^. The fact that even pigs kept indoors with concrete floors and metal fencing are driven to express ONF behaviors^4^ indicates that this is a set of behaviors that pigs are motivated to express, yet few EE devices accommodate ONF behaviors other than chewing. We have developed prototype EE devices that involve pigs pushing on a panel in a rooting motion. A video showing the operation of this EE device is in supplementary materials (S4). Pigs voluntarily root and push on the device that rewards them with a spray in their ONF regions. We examined if pigs would successfully self-deliver an oral vaccine in an efficacious manner by using the EE device to both cause pig interactions and elicit an antibody response. The labor savings if this technology works would be very large compared to hand-drenching and would allow for precise timing and dosing of sub-populations not currently achievable with current technology.

Successful oral vaccine delivery is commonly constrained by human error to the extent that manufacturers have devoted marketing and technical resources to how such errors can be avoided^5^. In commercial swine rearing, post-weaning oral vaccine administration is done via water systems which often require the entire population being supplied by the system to be vaccinated even if only a proportion of the pigs are at risk. Commercially available oral vaccines for swine are avirulent live cultures and are only viable for a few hours^6^. If certain pigs do not drink medicated water in that period, they may not be vaccinated. Individual pigs could miss becoming vaccinated with a self-vaccinating device, therefore, behavior data and antibody titers are needed to confirm both exposure to the vaccine and its effectiveness building antibodies. Hand delivery of vaccines to individual pigs is used on farms, but this method has a large labor requirement, and some pigs may get missed.

Our hypothesis was that if we could accommodate natural pig behaviors using a form of operant conditioning, we could let pigs self-vaccinate which would save labor and perhaps improve efficacy of vaccine delivery. We used the target species (domestic pigs) for this work, not a model species. This work was done using commercial pigs as are found on farms. The facilities and procedures are modelled after common practices on farms. The work is done using the target species in a setting that models modern, commercial pig production.

Our primary outcome measures were serum and oral fluid antibody levels after vaccination. A secondary but essential outcome was that pigs self-vaccinated themselves within the 6-h window of vaccine full viability. We report that pigs self-applied the vaccine using a modified EE device and that this self-vaccination resulted in successful detection of *Salmonella*-vaccine-induced IgG and IgA by means of an adapted a commercial enzyme-linked immunosorbent assays (ELISA) kit. This EE-vaccine system successfully delivered the *Salmonella* vaccine such that all pigs had *Salmonella*-specific IgG and IgA levels in their serum and oral fluids.

## Results

### Pig behavior

**Figure 1:**
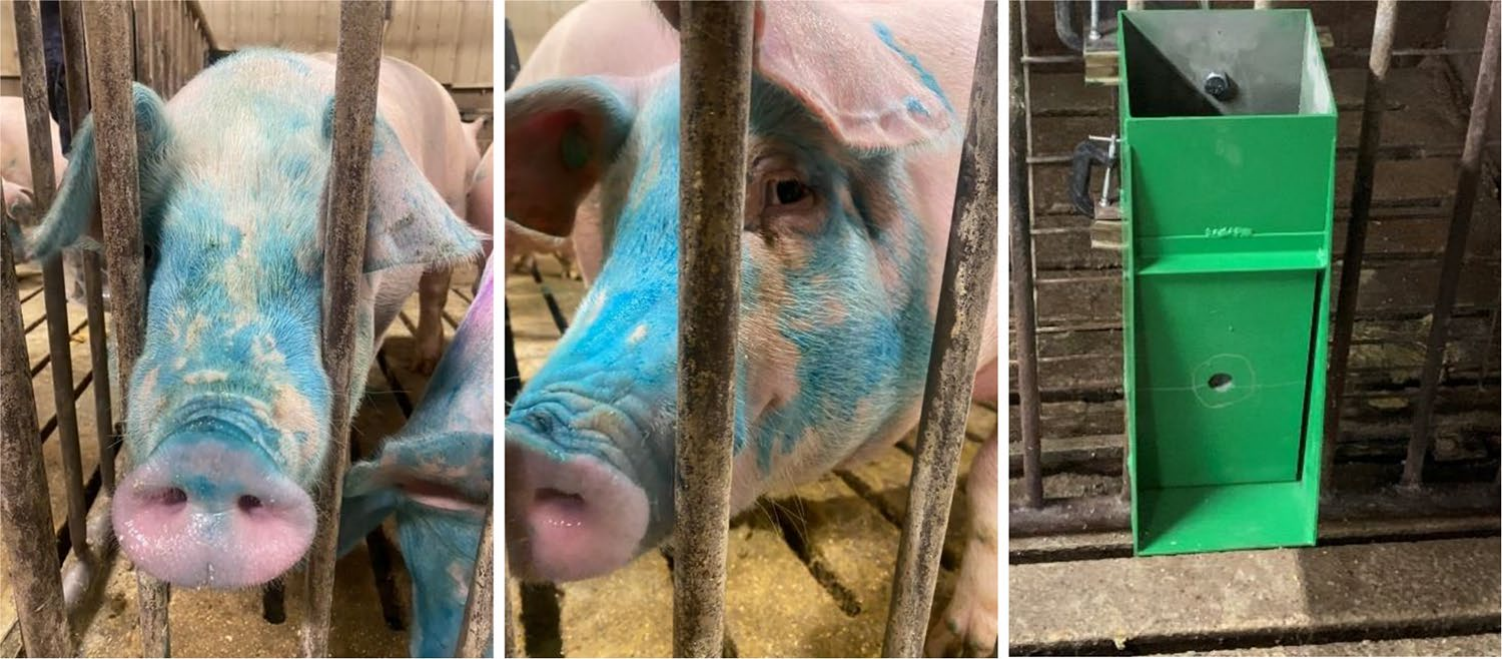
Example 4-month-old pigs self-vaccinated using an EE device and a picture of the sprayer device. Note the blue dye is added to the vaccine to identify that the pig has been exposed to the vaccine. Note also that the EE-device delivered vaccine to each pig’s nasal, oral and ocular mucous membranes. When a pig presses on the lower half of the front panel, a trigger is pressed, and 4 mL is delivered. This EE device in this form requires no external power or plumbing. A video of sprayer operation is given in Supplementary materials.

Pigs interacted with the self-vaccination sprayer delivering the oral *Salmonella* vaccine such that the blue dye made it easy to see where the antigen was self-applied to the face, eyes, mouth, and nostrils (Fig. 1). Vaccine could flow over the face and onto the ONF area to expose the mucous membranes and nasal cavity to the vaccine. For both the hand-drenched and self-administered treatment groups, the vaccine could be licked off the ground or from other pigs. From video recording of sprayer use, we determined that all the pigs in the self-administration treatment were sprayed within 80 min of exposure to the self-vaccination sprayer. Because the vaccine manufacturer claims viability for only 6 h after reconstitution, we confirmed that both the hand-drenched and self-administered pigs had exposure to the vaccine within the period of viability based on presence of the blue compliance dye being on the ONF area of each pig (Fig. 1).Pigs in these 2 treatment groups were effectively vaccinated (that is, they built antibodies to the antigens in the vaccine).

### Serology

**Figure 2:**
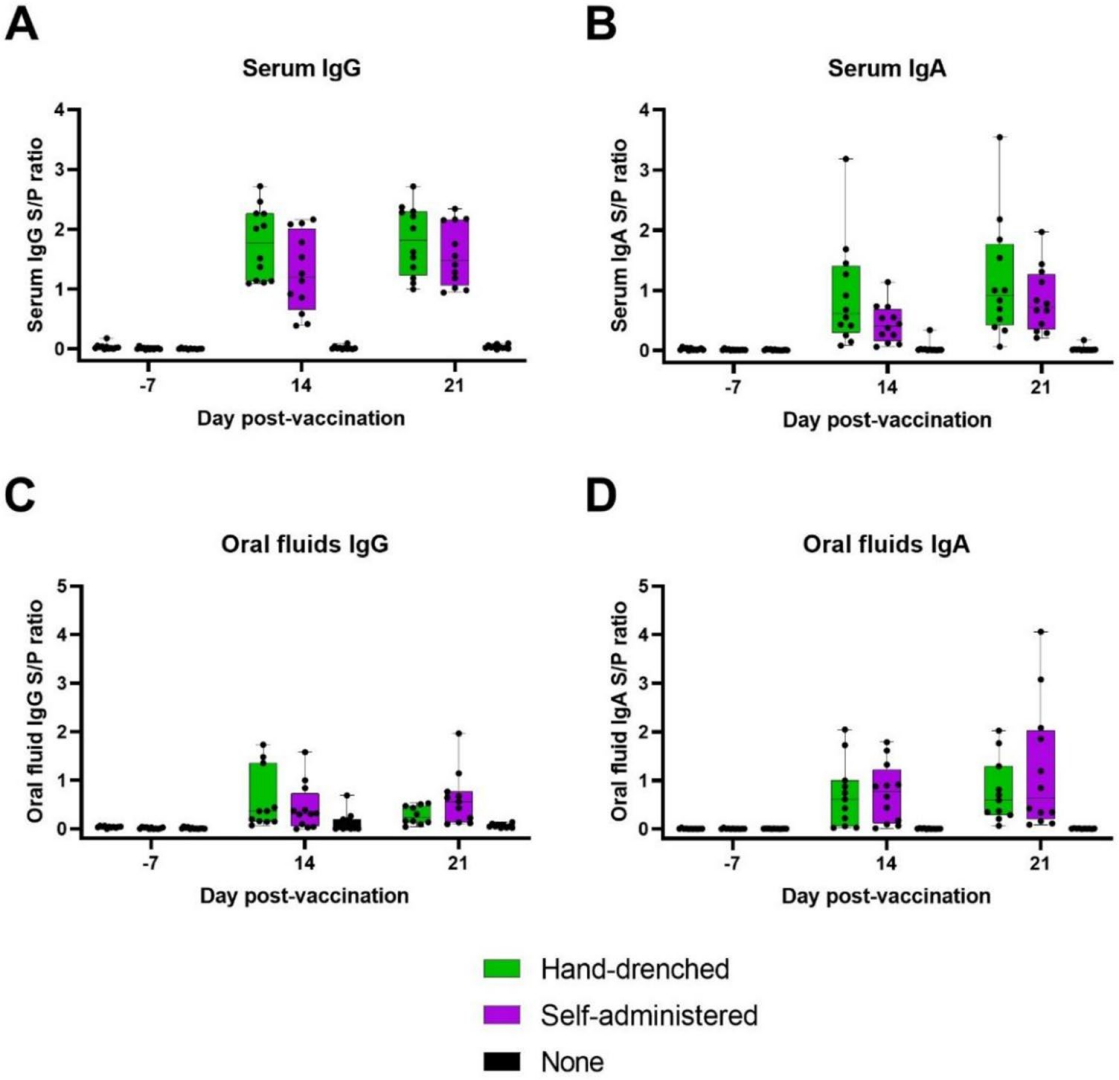
Graphic representation of the data showing individual values and the variation within treatments. Note that Control pigs (None) have little or no IgG or IgA at all times. Note also that IgG and IgA levels are statistically similar for pigs that were hand drenched or self-administered the *Salmonella* vaccine. Graphs represent the days peri-vaccination (days -7, 14 and 21) with panel A serum IgG, B serum IgA, C oral fluids IgG and D oral fluids IgA.

Pigs had variable background antibody IgG and IgA concentrations to *Salmonella* at time zero. Furthermore, we found heterogeneity of variance over time points that prevented evaluation by repeated measures analyses; therefore, treatments were evaluated at each time point (variances were homogenous within time points). Pigs that were in the hand-drenched or self-administration treatment groups had increased (P < 0.05) IgG and IgA serum and oral fluid concentrations at most time points (Table 1). At 14 days after vaccination, serum IgA and oral fluids IgG for the self-administration treatment had antibody levels that did not differ statistically from either the control or the hand-drenched groups. The variation in antibody levels across groups can be observed in Fig. 2. Evidence from video recordings confirms vaccine exposure of all 12 pigs in the self-vaccinated group. Overall, the data support the notion that 100% of pigs in hand- and self-administered treatment groups showed a vaccine-type response by 21 days after exposure to the vaccine.

**Table 1:** Serum and oral fluids relative concentrations of antibodies (S:P) to IgG and IgA for pigs in the three treatment groups.

Measure	Control	Hand drenched	Self-vaccinating	SE	P-value
Serum					
Day -7 IgG	0.010	0.033	0.009	0.009	0.14
Day 14 IgG	0.14^a^	1.58^b^	1.35^b^	0.170	0.003
Day 21 IgG	0.24^a^	1.81^b^	1.62^b^	0.160	< 0.0001
Day -7 IgA	0.013	0.021	0.012	0.004	0.14
Day 14 IgA	0.106^a^	0.804^b^	0.496^a,b^	0.155	0.026
Day 21 IgA	0.090^a^	1.060^b^	0.880^b^	0.190	0.006
Oral fluids					
Day -7 IgG	0.012^a^	0.038^b^	0.014^a^	0.005	0.005
Day 14 IgG	0.126^a^	0.660^b^	0.442^a,b^	0.170	0.13
Day 21 IgG	0.24^a^	1.81^b^	1.62^b^	0.200	< 0.0001
Day -7 IgA	0.006	0.007	0.006	0.002	0.94
Day 14 IgA	0.00^a^	0.793^b^	0.813^b^	0.167	0.003
Day 21 IgA	0.00^a^	0.864^b^	1.318^b^	0.270	0.007

N = 12 pigs per treatment mean unless there was insufficient quantity for testing. Least squares (LS) means within a row with a different superscript (^a,b,c^) differ, P < 0.05. For LS means on days 14 and 21, the time zero values were used as a covariate to equalize time zero values across treatments.

During descriptive assessment of the data, a non-vaccinated control pig (R261) housed in Pen 20 was identified as an outlier (This animal had a titer more than 2 standard deviations above the treatment mean); Supplemental information contains all data, including data from this animal). Inclusion or exclusion didn’t result in changes of observed treatment differences.

### Bacteriology

Diagnostic bacteriology was conducted on individual fecal samples from all pigs prior to vaccination. Additional fecal samples were collected throughout the study from which Salmonella sp. was not detected. This was done to rule out a wild-type challenge with *Salmonella* sp. during the study period that could be attributed to causing the antibody response. *Salmonella* sp. Was not identified in any fecal sample tested.

## Discussion

Both hand-drenching and self-administration of the vaccine was associated with significant elevation in IgG and IgA response in all pigs consistent with a vaccine-type antibody response. Pigs that self-administered the vaccine had equal or higher IgA in their oral fluids than did hand-drenched pigs 21 days after vaccination. We hypothesize that self-administration delivers equal or more antigen to more mucous membranes compared with hand drenching. Hand drenching directs the vaccine in each pig’s mouth. With self-vaccination, pigs expose multiple mucous membranes to the antigen most notably their eyes, nasal cavity along with their mouth (hand-drenched pigs do the same to some extent). In addition, self-vaccinated pigs expose themselves to multiple doses of antigen (they can spray themselves at will).

Self-vaccination relies on pig behavior to administer the vaccine. Risks of failure (inadequate antibody titers) include (1) some pigs might not get vaccinated, or (2) some pigs may receive multiple vaccine doses. Our behavior data and the resulting Salmonella-specific antibodies confirm that all pigs were vaccinated. Within 80 min all pigs had at least one dose of vaccine. In a commercial setting with more pigs per pen, the numbers of sprayers relative to the number of pigs will have to be titrated. We see minimal to no risk of over vaccination. Some pigs received multiple vaccine doses when the vaccine is added to water used by watering devices. No harm has been reported because of pigs receiving multiple vaccine doses from vaccines added to the watering system. Future systems may use tracking systems to only turn on the sprayer if a given pig has not been vaccinated. Hand drenching of pigs to deliver oral vaccines is a very time-consuming activity. The large labor requirement for individual pig handling and vaccination reduces the likelihood that such vaccines would be delivered this way if the farm does not have sufficient labor. If pigs could self-vaccinate, then this would significantly reduce labor needs and it would allow individual pens to be vaccinated to avoid vaccination of the entire herd through the drinking water system. This study demonstrated that self-vaccination triggered an IgG/IgA antibody-mediated response, detected in serum and oral fluids, which did not differ from vaccination performed by hand drenching.

Despite previous unsuccessful attempts towards adapting commercial Salmonella ELISAs for antibody detection in oral fluids^7^, the methodology presented here demonstrates that indirect ELISA can be used to detect *Salmonella* IgG and IgA antibody responses at pen (based on oral fluids) and individual pig (serum) levels. In the present study, the IgG response was higher in serum while the IgA response was higher in oral fluids. Similar findings were reported for porcine epidemic diarrhea virus (PEDV) by Bjustrom-Kraft^8^. Different studies demonstrated that serum and oral fluid IgG and IgA antibody kinetics vary among swine pathogens^8–10^. IgA is the most abundant class of antibody in intestinal secretions and mucosal surfaces and prevents Salmonella from invading intestinal epithelial cells^11^. Whereas IgG, the most abundant antibody isotype in blood (plasma), is required for the clearance of Salmonella in the late phase of the primary infection, and it is critical for the enhancement of phagocytosis during secondary immune responses^12^.

While serum remains a reliable sample for diagnostic evaluation, collection of oral fluids is widely practiced as it requires minimal skill or pig handling by employees and provides a suitable matrix for detection of pathogen-specific antibody and nucleic acid^11^. This sprayer causes pigs to leave oral fluids as they are manipulating the device. Minor adaptations to the sprayer would allow any-time collection of oral fluids as an aide to diagnostics.

We believe that any new technology should be assessed for their impact on animal welfare. Because the vaccine caused no harm and might have prevented disease, there is no negative animal welfare associated with this technology.

Before we started, we did not know the adequate sample size to detect meaningful differences. We now know that our sample size was adequate to detect the differences that were observed. Future work could determine antibody decay following vaccination or infection, and the extent to which IgA in oral fluid contributed to mucosal immunity and disease outcome.

This technology can be used for other animal health products. Pigs would use an EE device such as the one we developed to self-apply hormones, pheromones, other oral vaccines, intranasal vaccines, oral antibiotics, or other animal health products. Pigs were encouraged to interact with the sprayer by including the maternal pheromone which increases interest by the pigs^13^. The novelty of the sprayer also entices exploration which is a desired feature of EE devices^14^. Pigs manipulated the sprayer at a low level every day even if the reward was just water or the movement of the pushing plate. By adding the maternal pheromone, pigs increase their interest in the device. Use of vaccine stabilizer containing a blue compliance dye (Reload Pack) was done per manufacturer instructions in addition to the maternal pheromone. Adding these elements to the vaccine dose did not impact vaccine viability or antibody synthesis.

The device we tested was mechanical and had no electronic or plumbing parts. This device could be installed to treat individual pens of pigs (or other animals) rather than mass treatment. Considering not just vaccines can be delivered, use of this technology could all spot treatment with antibiotics or other animal health products. The simple self-administration device could also be of value to smaller farms or in more remote areas where power is either not available or expensive.

As a part of newer smart-barn systems, the sprayer could be plumbed and have electronic valves so that different liquids (or powders) could be added and operated remotely. This means that in the near future when wired and plumbed EE devices are in each pen, a person could sit in an office and treat as many pens as needed with any given animal health product with a high degree of compliance. By accommodating the behavioral need of pigs to root and push, every pig with access touched this EE device (usually several times). While interacting with the EE device, pictures of the animals can be taken and analyzed to capture the current health status and welfare of individual animals.

In this work, we used a live oral vaccine. We do not yet know if this technology will work for killed vaccines. Nor do we know if this technology will work for intranasal vaccines or those given as an intramuscular injection. We believe we can modify this sprayer to allow for delivery of other types of vaccines, but this will have to be confirmed in future engineering and experimentation.

Feral swine are a hazard to the environment and commercial pig producers. This device and associated technologies could be used by feral swine to vaccinate or medicate themselves or to selectively deliver reproductive sterilant. Having oral fluid samples for commercial or feral swine would allow more comprehensive surveillance of infections including zoonoses like Salmonella or diseases like African swine fever virus (among many others).

In conclusion, we demonstrated efficacy of a device to allow pigs to self-administer an oral vaccine and induce a vaccine-type antibody response. We also adapted testing for isotype-specific immunoglobulins which will assist in more timely and accurate *Salmonella* serodiagnosis. This device and associated technologies will help pork producers better manage animal health while providing environmental enrichment with less labor. In addition to commercial, wild and feral swine, this concept can be applied to any species of farm or wild animal. The device would have to be modified to accommodate the behaviors of the target species, but the basic concept could potentially be used with any species.

## Materials and methods

The specific objective was to evaluate three treatment groups to test the efficacy of self-administration as a means of vaccine delivery: (1) Control – no vaccine exposure, (2) Vaccinated by hand drenching as per manufacturer’s instruction, and (3) Pigs self-vaccinated using a novel EE device.

### Animals and experimental design

This study and this paper are consistent with ARRIVE guidelines 2.0. This report adheres to both the ARRIVE essential 10 and the Recommended Set of additional reporting (https://arriveguidelines.org/). All methods were performed in accordance with local and federal laws and guidelines and international standards. The protocol was approved by Texas Tech University Institutional Animal Care and Use Committee (IACUC #22,020-03 March 7, 2022). Texas Tech University’s animal care program is fully accredited by AAALAC International, and the university program is inspected by the USDA for compliance. The IACUC reviews the protocol which must be approved before the work started. We do not have a local mechanism to register our protocol. Because this work is novel, no existing protocol for the animal work has been reported. All data will be available in supplementary materials linked to this paper.

The work was conducted at the Texas Tech University Swine Unit which is a smaller-scale modern commercial farm with slotted concrete floors and mechanical ventilation. Each pen has hanging chains for environmental enrichment (EE). However, for pigs with the sprayer, they would have had additional EE because they interact with the sprayer willingly. Because of the nature of the vaccine, pigs did not become ill. No pig illness or deaths were observed. No adverse events were noted. Several weeks after the end of the study, pigs were marketed as commercial pigs, that is, they were sold to a broker who sold them to a slaughter plant.

The animal work was performed at Texas Tech University (TTU, Lubbock, TX USA). The laboratory assays were performed at Iowa State University Veterinary Diagnostic Laboratory (ISU VDL, Ames, IA USA). It was not possible to blind TTU scientists because of the nature of the study and the obvious pig treatments in each treatment group received. Each treatment group was obvious to even a casual observer. However, the samples were sent to ISU with unique labels that allowed ISU scientists to be blinded to treatment groups. All laboratory analyses were performed with collaborators blinded to treatment groups.

All pigs were PIC genetics with a white-line sow and a Duroc boar. This is a common genetic line of commercial pigs. Pigs had access to a corn-soy based commercial feed and water ad libitum. There were three pens per treatment, each individual pen housed 2 castrated males and 2 females (12 pigs per treatment and 36 pigs in total). Pigs were 15 weeks of age at the start of the study which was an age and weight about 2 months before slaughter (about 4 months of age). Each pig was identified at birth with a unique alphanumeric colored ear tag. Pigs were randomly assigned to pens and pens were randomly assigned to treatment group (however, after moving pigs/pens, treatment pens were clustered to avoid cross-contamination among treatments). Pigs were not blocked on weight or pre-vaccination antibody levels because they had previously spent 12 weeks with their pen cohort and mixing would result in their need to re-establish social hierarchy (so the stress of mixing was avoided).

Average starting weights were different between treatment groups necessitating inclusion of weight as a covariate in the analysis (S3). All pens were in the same barn. Each treatment group was accommodated in a block of three adjacent pens, with nose-to-nose contact possible through fencing within a treatment group (S2). An empty pen or aisle was placed between treatment blocks or contemporaries not enrolled in the study to prevent nose-to-nose contact between treatment groups (S2). Pens were clustered by treatment assignment because the avirulent live vaccine can be shed for a minimum of 4 weeks following vaccination.

Control pigs received no vaccine or treatment. Hand-drenched pigs received the vaccine per os as instructed on the label with a person following the pigs and placing the vaccine in their mouth. For potentially self-vaccinating pigs, One sprayer was placed in each of the 3 pens for a maximum of 5 h. After consumption, the reservoir was filled with water and left in the pen for an additional 18 h. Based on previous observations, 15-week-old pigs exposed to the sprayer for the first time would visit 2 times per hour. Therefore, a 200 mL total volume was placed in each sprayer reservoir at the ratio of 10 mL of vaccine (equivalent to 1.25 doses per pig), 1 mL of maternal pheromone^13^ to encourage active engagement with the sprayer and 189 mL of sodium thiosulfate treated water.

Three pens housing 4 pigs each were assigned to receive vaccine via an adjustable height pen-mounted prototype EE device that pigs could operate by pressing a panel with their snout (Fig. 1, S4). When pressed hard enough the panel triggered a spray with up to 4 mL delivered from the sprayer reservoir. Based on previous observations, 15-week-old pigs exposed to the sprayer for the first time would visit 2 times per hour. Therefore, a 200 mL total volume was placed in each sprayer reservoir at the ratio of 10 mL of vaccine (equivalent to 1.25 doses per pig), 1 mL of maternal pheromone^13^ to encourage active engagement with the sprayer and 189 mL of sodium thiosulfate treated water. One sprayer was placed in each of the 3 pens for a maximum of 5 h. After consumption, the reservoir was filled with water and left in the pen for an additional 18 h. Apart from the addition of the maternal pheromone attractant, the hand-drenched and self-vaccinated pigs received.

### Statistical analyses

The pig was the Experimental Unit because treatments were applied to the individual pigs. Twelve pigs were sampled per treatment. The sample size could not be estimated based on past work because such studies have not been reported. We used professional judgment to come up with a sample size of 12 pigs per treatment group for the 3 treatment groups.

The study was conceived as a completely random design with a split plot over time (3 time points: day -7, 14 and 21). Groups had an equal number of pigs (no morbidity or mortality were recorded) in each treatment. An examination of the data showed heterogeneity of variance among time points, making the full model not valid. Therefore, we analyzed treatment effects at each time point. At time zero (7 days prior to vaccination), some pigs had background antibody to Salmonella. Thus, for time points 14- and 21-day post vaccination (dpv), the time zero antibody levels were included as a covariate to equalize background antibody at time zero. A few oral fluid samples had inadequate volume for analyses. Thus, General Linear Models was used to evaluate treatments. The statistical model included three treatments (Control, Hand-drenched, and Self-vaccinated) at time zero and then the same model with the added time zero values and starting body weight as covariates. Within each time point, Least Squares means were separated using the Predicted Difference test within SAS Studio software (Release 3.8, 2020). The raw data and an example statistical analysis is provided in supplementary materials (S5).

### Vaccine handling and administration

We used a commercial, lyophilized, bi-valent avirulent live *Salmonella typhimurium*-*Salmonella choleraesuis* vaccine culture (Enterisol Salmonella T/C, Boehringer-Ingelheim Animal Health, USA). The vaccine label states use “aids in the prevention” of *S. Cholerasuis* and *S*. *Typhimurium*”; therefore, generation of antibodies is expected as an outcome. The vaccine was stored and handled per manufacturer recommendations (supplemental information, S1). Once reconstituted, the vaccine was then added to distilled water containing sodium thiosulfate and blue dye (Reload Pack) mixed at a ratio of 1 bottle to 3.8 L to aid in the administration of oral vaccine. To maintain vaccine culture viability, vaccine was added to drinking and stock solution water treated with Reload Pack and consumption was complete within 6 h of reconstitution. For pigs in the sprayer pens, the maternal pheromone was added at 10 ppm which we have shown increased pig interest^14^. After vaccine preparation, each pig was exposed to at least 2 mL of vaccine (manufacturer’s labeled dose; Enterisol Salmonella T/C, Boehringer Ingelheim Animal Health USA Inc.) added to a larger volume of liquid to facilitate administration as appropriate for the delivery method.

The pigs in the 3 pens comprising the hand-drenched group received a 5 mL total volume at the ratio of 2 mL vaccine and 3 mL sodium thiosulfate treated water. The specified volume per pig was aseptically drawn into a plastic dose syringe. Without restraining pigs, the syringe was inserted into the corner of each pig’s mouth and vaccine was administered per os. For hand-drenched delivery, one author (RCR) administered the vaccine and was careful to be sure a dose was delivered in each pig’s mouth and that pigs in the self-vaccination and control groups were not contaminated.

### Feces, serum, and oral fluid collection

Individual fecal samples were collected from individual pigs as it was passed or using a gloved finger to obtain at least 1 g feces per pig. Restraint was not required in order to collect feces. Individual pig specimens were submitted 7 days prior to vaccination (time zero) from all pigs. Samples from the sprayer group were tested individually on 1, 3, and 7 dpv. Samples from the drench and control groups were individually collected then pooled by pen for testing on days 1, 3 and 7 dpv. At 14 dpv, feces were collected from individual pigs then pooled at the farm by pen for each pen. Finally, a fecal composite collected from each pen’s dunging area was collected at 14 and 21 dpv.

Blood samples were collected from the cranial vena cava. Pigs were restrained as appropriate for their age of production for blood collection using a hand-held hog snare. Blood was collected 7 days prior to vaccination (time zero) then 14 and 21 dpv. Blood was spun at 2500 rpm for 10 min in a bench-top centrifuge. Serum (3–5 mL) was transferred to leak-proof tubes for transport to the AAVLD-accredited veterinary diagnostic laboratory for antibody testing.

Oral fluids were collected using cotton balls tied to a string. Individual pigs were allowed to chew on cotton balls until moistened. Samples were collected 7 days prior to vaccination (time zero) then 14 and 21 dpv. Moist cotton balls were wrung out into plastic bags to obtain 1–2 mL then transferred to plastic screw cap tubes (1 to 2 mL) for transport to the AAVLD-accredited diagnostic laboratory serology section for antibody testing.

All samples were placed on ice or ice packs immediately after collection. The diagnostic laboratory best suited to perform testing was in another state. Therefore fecal, serum and oral fluid samples were shipped overnight on ice packs in a Styrofoam cooler to minimize temperature insult. Feces were shipped to the diagnostic laboratory within 18 h of collection. Serum or oral fluids not shipped within 12 h of collection were stored in an upright freezer at -20 °C.

### *Salmonella* bacteriology

Feces were placed into a sterile leak proof container for transport. Each sample was cultured for *Salmonella* under selective enrichment media (tetrathionate) broth and selective/differential agar plates following standard protocols at the AAVLD (ISU VDL).

### *Salmonella* IgG/IgA indirect ELISA

A commercial *Salmonella* serum blocking ELISA (IDEXX Swine *Salmonella* Ab Test) was adapted into an indirect ELISA format (i.e. sample dilution, assay conditions, conjugate, substrate, and stop solution) for isotype-specific antibody (IgG, IgA) detection in serum and oral fluids. In brief, serum samples were tested at 1:20, while oral fluid samples were tested at a 1:2 dilution using kit sample diluent (100 µL final reaction volume). After 1 h incubation at 37 °C, microtiter plates were washed 5 times (350 μL/well) with kit washing solution, and 100 μL of corresponding conjugate was added to each well and incubated at 37 °C for 30 min. Specifically, the kit conjugate was replaced with peroxidase-conjugated goat anti-pig (Fc) IgG (Bethyl Laboratories, Inc., Montgomery, TX, USA) at 1:40,000 (serum) or 1:3000 (oral fluid); or peroxidase conjugated goat anti-pig IgA (Bethyl Laboratories, Inc.) at 1:5000 (serum/oral fluid) in conjugate diluent (20% fetal bovine serum, 0.05% Tween 20, phosphate buffered saline, pH 7.4). After another washing step, the reaction was visualized after 5 min incubation with 100 μL of tetramethylbenzidine-hydrogen peroxide (TMB) substrate solution per well (Surmodics IVD, Inc., Eden Prairie, MN, USA) and stopped with 100 μL of stop solution per well (Surmodics IVD, Inc.). Optical density was measured at 450 nm using an ELISA plate reader and SoftMax Pro7 software (Molecular Devices, Sand Jose, CA, USA). Antibody responses were expressed as sample-to-positive (S:P) ratios.

## Supplementary Information

Supplementary Information 1.

Supplementary Information 2.

Supplementary Video 1.

## Data Availability

The complete dataset and an example analysis is available in supplementary materials.
