# Supplementary material for: Self-administration of a Salmonella vaccine by domestic pigs
Source: Sci Rep. 2023 Feb 20;13:2972. doi: 10.1038/s41598-023-29987-x (PMC9941462; doi:10.1038/s41598-023-29987-x)
Supplement: Supplementary file 2 — Supplementary Information 2. [file 41598_2023_29987_MOESM2_ESM.docx]

Supplemental Information

Supplementary Information 1. Vaccine manufacturer information

Salmonella vaccine from Boehringer Ingelheim (Duluth, Georgia, USA). https://www.bi-vetmedica.com/sites/default/files/dam/internet/ah/vetmedica/com_EN/product_files/Enterisol%20Salmonella/EnterisolTC_label.pdf

Supplementary Figure 2. Pen diagram (not to scale)


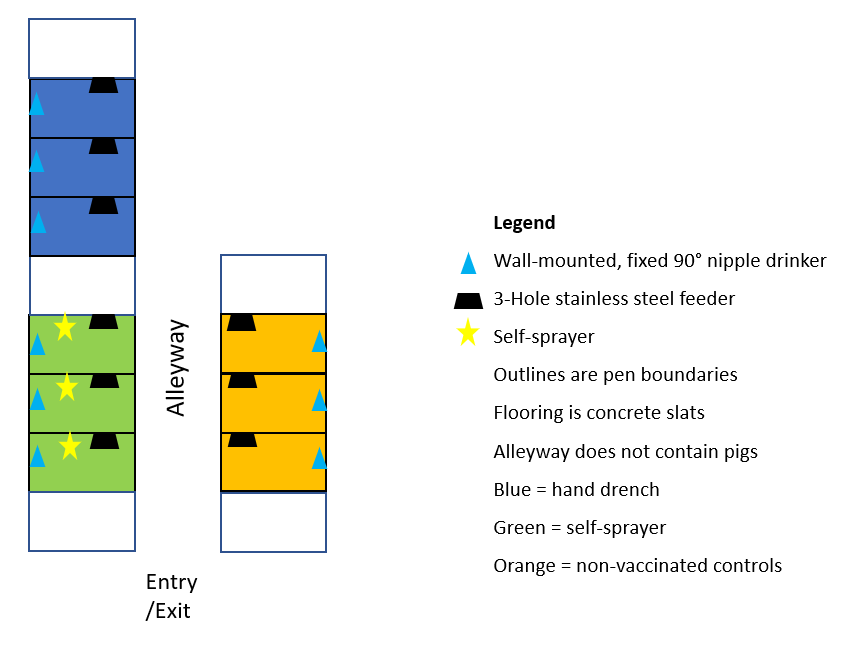


Supplementary Table 1. Starting, ending weights and average daily gain of pigs by treatment group. Pigs and pens were randomly assigned to treatments. Time zero body weights for Control pigs were lower (P < 0.05) than pigs in the hand drenched group. ADG and body weights 45 days after the initial body weights were not significantly different (P > 0.05) among treatments.

|  | Average pig body weights*, Kg | | Average daily gain |
| --- | --- | --- | --- |
| Treatment | Wt 0 | Wt 45 | Kg/d |
| Control | 78.5^a^ | 132.2 | 1.19 |
| Hand drenched | 91.2^b^ | 139.7 | 1.10 |
| Self-vaccinated | 86.8^a,b^ | 130.6 | 0.97 |
| SE | 3.38 | 4.52 | 0.009 |
| P-value for treatments | 0.04 | 0.32 | 0.24 |

* Weights are indicated at time zero (Wt 0; 18 days before vaccinating and 45 days later (Wt 45) which is 3 weeks after vaccination.

^a,b^ Least squares means with different superscripts within a column differ, P < 0.05.

Supplementary Video 1. Videos of (1) the EE device operation and (2) pigs self-administering the vaccine.

Supplementary Information 2. Raw data and an example statistical analysis code. These data are provided in a spreadsheet that is provided.
